# Elderly Cancer Survivors Reflect on Coping Strategies During the Cancer Journey

**DOI:** 10.4172/2167-7182.1000337

**Published:** 2016-08-19

**Authors:** Eva Kahana, Boaz Kahana, Kaitlyn Barnes Langendoerfer, Baruch Kahana, Alicia Smith-Tran

**Affiliations:** 1 Department of Sociology, Elderly Care Research Center, Case Western Reserve University, Cleveland, OH, USA; 2 Cleveland State University, Cleveland, OH, USA; 3 Marshfield Clinic, Wisconsin, USA

**Keywords:** Elderly, Cancer, Traumatic, Older

## Abstract

**Purpose:**

This paper focuses on perspectives of elderly cancer survivors on their experiences of coping with cancer during various phases of their illness journey, ranging from diagnosis phase to treatment and finally considering post treatment survivorship. Anchored in the stress paradigm, the purpose of this study was to explore the meaning of living with cancer and older adults’ orientations to coping with stressors encountered during their cancer journey as reflected in narratives of elderly cancer survivors.

**Methods:**

A nonclinical sample of 174 older adults who reported a cancer diagnosis were selected from a panel study of successful aging. In-depth interviews with respondents focused on perceived stressors and coping strategies at different phases of their cancer experience. Themes were derived from narratives based on consensus by two raters.

**Results:**

Most of the elderly patients accepted their diagnosis without dismay. Resolve and determination during the diagnosis phase was followed by assuming a more passive role during the treatment phase, relying on expert medical care. During the longer term survivorship phase older adults looked back at the adaptations they found most useful. The majority reported valuing active coping styles. These include seeking social support and instrumental orientations to dealing with the illness followed by religious or spiritual approaches.

**Conclusions:**

The “on time” interpretation of having a cancer diagnosis in old age may diminish the stressfulness of the diagnosis and may enhance the patients’ ability to proactively deal with the reality of their illness.

## Introduction

This study focuses on elderly cancer survivors’ perspectives of their illness journey. Specifically, the purpose of this study was to explore older patients’ characterizations of salient aspects of coping during their time as patients and survivors [1]. Our study utilizes a qualitative approach to capture the coping strategies of patients through the lens of survivorship. Anchored in the stress paradigm, this study examines the meaning of living with cancer as reflected in the accounts of elderly cancer survivors [2].

Previous literature has framed cancer as a traumatic stressor while focusing specifically on strategies of coping among younger patients [3]. Our study is unique, as it looks at the cancer journey in terms of different phases of illness (diagnosis, treatment, and survivorship), with a focus on the experiences of older adults. The term “coping” generally describes a response or strategy that individuals use to manage problem situations and to protect themselves from potential adverse consequences of stressful life events [4]. Coping strategies encompass appraisals of stressful life situations as well as cognitive, behavioral or emotional responses to the stressors [5]. Prior research points to strong negative emotional responses by cancer patients who are facing an uncertain future [6,7].

In this study, we sought a more nuanced understanding of coping with a life threatening illness in old age as defined by the respondents. Specifically, we wanted to learn about the differentiation of coping responses as challenges shift during different phases of the cancer experience.

## Background

Extensive literature has focused on coping strategies of younger cancer patients in dealing with various cancers [8,9]. However, there is limited scholarship available on the perspectives of older adults toward the challenges and realities of coping with cancer during various phases of the illness. Older adults have been underrepresented in cancer research as well as in treatment trials, despite an increase in the number of older people with cancer [10,11]. Even though older cancer survivors may not face the same social stressors associated with initial diagnosis and treatment as their younger counterparts (e.g. loss of a job, fulfilling parenting roles), they still face stress related to enduring symptoms related to the illness and its treatment and the fear of reemergence of the disease [12,13].

We recognize that the adaptive tasks that cancer patients face vary during the different phases of one’s illness journey [14]. For example, the need to seek out information tends to be particularly acute during the diagnosis phase, when patients have to ascertain their treatment options. Accordingly, managing the diagnosis of cancer calls for very different cognitive and behavioral adaptations then does the treatment experience and the experience of long-term survivorship [15]. Furthermore, the diagnosis and treatment phases are relatively short-term whereas survivorship covers a far longer time span.

Receiving a diagnosis of life-threatening illness, such as cancer, represents a potentially traumatic stressor. The challenge of a cancer diagnosis has been described in the literature as a crisis, a turning point and as a confrontation with death [16]. The diagnosis phase has been considered as one of the most difficult stressors encountered by the cancer patient [17]. Being diagnosed with cancer can create uncertainty that shatters assumptions of invulnerability that most healthy individuals live with [18]. Stressors encountered by the patient include symptoms of illness that prompted the search for a diagnosis, the emotional distress brought about by the diagnosis and the discomfort and challenges posed by further diagnostic tests and treatments. Additionally, concern about understanding treatment options and making wise decisions about them may be uppermost in patients’ minds during this phase of the illness journey.

The treatment phase is typically shortly after the cancer diagnosis. During this phase, patients are often focused on the potential risks of invasive procedures and adverse treatment effects [12]. Consequently, the quality of communication with physicians and other health care staff assumes great importance as patients tend to be interacting with their health care providers more frequently during this phase [19]. Indeed, management of communication emerges as a salient coping strategy [15].

Following the treatment phase, researchers have found that some patients become concerned with fears of recurrence [20]. The transition from being a cancer patient who is in active treatment to a cancer survivor who is no longer closely monitored by health care professionals has been shown to be a challenging transition that can bring with it uncertainty and a feeling of lack of support [11]. Deimling in 2006, found that older patients who made a successful transition to perceiving themselves as a “cancer survivor” had higher self-esteem and well-being as they were less fearful about reoccurrence of their cancer [12].

The long-term sequalae of having experienced life threatening illness can cast a shadow on long-term well-being among survivors [21]. Studies comparing psychosocial adjustment of community dwelling cancer survivors with adults who did not experience cancer reveal greater psychological distress among cancer survivors than their counterparts who did not have a cancer diagnosis [22]. However, it is notable that older survivors were found to be more resilient and to portray greater well-being than their younger counterparts. Moreover, there are indications that long term cancer survivors engage in and prefer optimistic, supportive, and proactive strategies [23].

It is important to note that there is some controversy in this, primarily within the quantitative literature, regarding vulnerability vs. resilience in the aftermath of surviving cancer. Our research sought to shed light on this controversy by offering more qualitative insights based on accounts of elderly survivors who can look back at different phases of their cancer experience and recount their experiences as well as their coping efforts. We hope that our study will broaden understandings about the lived experience of cancer reported through the prism of old age and add useful understandings to cancer survivorship research [24,25].

## Methods

### Sample

Our sample was drawn from a longitudinal panel study of successful aging with 1,450 community-dwelling older adults [26]. As a follow-up to the initial survey study, separate interviews were conducted with respondents who reported a previous cancer diagnosis (N=174). Our sample is unique, based on its inclusion of a nonclinical sample of community dwelling cancer survivors. Furthermore, the meaning of diagnosis and treatment are filtered through the prism of survivorship rather than conveying immediate reactions during the experience.

### Procedures

During the 60 to 90-minute in person interviews, the respondents were probed for descriptions of their experiences with different phases of their cancer journey, ranging from diagnosis to treatment and then transition to post-treatment (survivor) status. Open-ended questions focused on reactions to diagnoses, communication with the healthcare team during treatment and reflections on coping strategies patients employed to manage stressful life situations.

Two separate co-authors coded the responses to these questions independently as recommended by Basit in 2003 [27,28]. They met on several occasions to discuss any questions about their coding and to compare their independently coded data. In instances where the coders differed (<5% of the data), they discussed their interpretations and came to agreement. A total of 174 interviews were coded to derive themes commonly articulated by elderly respondents during each phase of their cancer journey. Descriptive statistics were also run using SPSS 22 to calculate percentages, means, and Standard Deviations (SD) of demographic data and close-ended questions [29].

## Results

The mean age of our sample was 81 years (SD=7.5) with 64 percent of the respondents being female. The overall sample was predominantly white (89%), with 11 percent being African-American. The majority of respondents (55%) were married, 35 percent were widowed and the remaining 10 percent were not married (i.e., never married, divorced or separated). Regarding education, 13 percent of respondents had less than 12 years of education, another 27 percent were high school graduates, 35 percent obtained 1 to 2 years of college or technical training, 13 percent were college graduates, and 10 percent did postgraduate work.

The respondents varied in terms of the site of their cancer (e.g. prostate, lung, and breast). The most common cancer diagnosis among respondents was breast cancer (27.8%), followed by prostate (17.2%), melanoma (14.6%), colorectal (10.6%), bladder (8.6%), gynecologic (6.6%), lung (6%), hematologic (5.3%) and kidney (3.3%). Just over one-third (36%) reported having had multiple cancer diagnoses. Referring to their most recent cancer experience, respondents reported a mean of 9.86 years (SD=11.42) since receiving their cancer diagnosis.

In the next sections, we discuss themes that emerged based on respondents’ reflections on the three phases of their cancer journey: 1) diagnosis; 2) treatment; and 3) survivorship.

### Diagnosis phase

Respondents provided various reflections of their experiences upon receiving the cancer diagnosis. Their ability to provide such in depth descriptions years after the diagnosis suggests that being diagnosed with cancer was a sentinel event in their lives. Some respondents reported they had a calm acceptance of their cancer diagnosis and recounted that they expected the diagnosis based on their age, family history, or symptoms. Others articulated shock, fear and confusion in response to their cancer diagnosis. As one woman stated, “I went to pieces and told him [the doctor] I wanted to die.” Another said, “I was upset- I thought why me?”

Although worry is considered to be an important emotional response by patients newly diagnosed with cancer, respondents in our study seldom articulated experiences of worry [12]. Their “on time” interpretation of having a cancer diagnosis may have diminished the stress of the diagnosis [30]. For instance, a respondent stated, “It was a little worrying, but at my age it really doesn’t matter.” Others realized that their cancer was caught early or that their treatment options were not invasive. One woman explained, “It was no big deal. I knew it is easily treated if caught early.” Another stated, “My doctor told me it was cancer and that is what I expected to hear. My reaction was okay this is not going to kill me.”

Respondents in our study were also asked how they felt after their initial reaction to their diagnosis. Their responses provided us with some insight into how patients accepted or assimilated the diagnosis into their lives. In other words, it gave us a sense of how they coped with their cancer diagnosis. When asked, “To what extent did you feel depressed following your diagnosis?” only 7.5 percent reported “a great deal” while 51.7 percent reported “not at all.” In terms of feeling emotionally numb following a diagnosis, only 8.6 percent said “a great deal” while 56.3 percent said “not at all.” Feeling determined was the most often reported orientation following a diagnosis. Over 93 percent of participants reported some extent of determination towards their diagnosis (ranging from “a little” to “a great deal”). Twenty-one of our participants went on to express other feelings following their diagnosis. One theme that emerged from this group was acceptance. One respondent illustrated this orientation by saying, “I did not worry about other people telling me how bad they feel on my behalf, I accepted my situation and just lived with it.” Others noted their anxiety toward the unknown. For example, some respondents expressed their fear of dying, disappointment, and anger with their situation.

### Treatment phase

The treatment phase of the cancer journey follows the diagnosis phase for those who have a cancer that has treatment options. The majority of respondents reported being passive in help seeking during the treatment phases, but described satisfactory support from both family members and physicians. Major frustrations related to the lack of input or choice in treatment decisions and long wait times between diagnosis and treatment. Nevertheless, most respondents reported maintaining a positive attitude and having overall satisfaction with physician communication and medical care.

Managing communication with the healthcare team was a salient aspect of the treatment phase of the journey for many of our respondents. Thus, we asked both open-ended and closed-ended questions regarding patients’ communication with primary care physicians, surgeons, medical oncologists and radiation therapists over the course of their treatment. Specifically, respondents were asked to describe examples of good communication, instances when they were excluded from the treatment decision-making process and times when there was a problem in communicating with the healthcare provider during the treatment phase of the cancer journey.

Our findings revealed mixed results for communication patterns between elderly patients and their healthcare providers during the course of their cancer treatment. When asked, “During your cancer treatment, to what extent was there good communication with your health care provider?”, only 35 percent reported that there was “a great deal” of good communication in comparison to the 34 percent that reported “not at all.” Even though there were differing results toward “good communication” with providers, only 4 percent of participants reported that they experienced problems with communicating with their health care providers during the treatment phase.

Respondents provided several different examples as to why they labeled their communication with their doctors as positive. One theme that emerged in terms of good communication was that the doctor provided them with educational information about their treatment. For instance, one respondent said, “The urologist gave me a book containing all the info I needed about the cancer and treatment,” while another stated, “When I asked them about physical limitations, they provided statistical data as a reference.” Other respondents felt that they communicated well with their physician based on receiving emotional support from their provider and feeling as though the provider listened to their concerns: “any time I had a problem he was supportive” and “they listened to my concerns and supported me.”

Respondents also reported that their communication was good if they felt they received personalized care. One respondent said, “The internist began the research of my unrelated back pain with many x-rays. This allowed my diagnosis to be made.” Another stated, “The doctors were readily available by phone. I believed the doctors answered any and all questions no matter how trivial or unrelated.” Respondents also expressed many different reasons for having experienced negative communication with their providers during the treatment phase of their journey. Common themes involved lack of communication all together (e.g. “I was not told anything about hot flashes”; “My surgeon was non-communicative. No one told me to go to an oncologist”). Patients also reported unwillingness of physicians to listen to their preferences (e.g. “I have bad veins and they wouldn’t listen to me how to take my blood”; “They wanted to do more to me than I wanted”). Finally, some respondents indicated that the provider was negligent. For example, one respondent stated that, “the first pulmonary MD misdiagnosed me. He told me it was not malignant and I waited a year before second opinion. It was much worse by then”. Another said, “We told him twice the spot was changing and he said it was OK. We insisted on biopsy and it was melanoma.”

### Reflections on the cancer journey during the survivorship phase

For most of our respondents, the survivorship phase spans a longer period compared to the initial diagnosis and treatment phases, which are typically compressed in a one or two-year time span. During this period, patients can take a broader look at evaluating their cancer journey, looking back at the course of their experience. Accordingly, we elicited patient perspectives on their overall coping approaches. We sought to learn their perspectives about the most successful coping strategies in dealing with the illness.

Analysis of responses yielded eight different coping categories that were derived from the narratives of 118 respondents who provided an answer to this question during the interview. It is noteworthy that 37 individuals offered multiple coping strategies. This reflects a recognition that a combination of strategies may need to be employed to effectively manage adaptive tasks posed by the cancer experience. We considered the types of adaptations described by our respondents as most helpful in dealing with their cancer experience in the context of coping styles described in the stress literature.

Indeed, the categories that emerged from our data include instrumental, intra-psychic, escape-oriented and emotional coping orientations [31]. The prevalence of different coping styles underscores the value placed by older adult cancer survivors on the specific instrumental strategies of marshaling social support on the one hand and religious coping styles on the other. For this sample of survivors, emotional expression was not a common coping strategy. It is also noteworthy that benefit finding and posttraumatic growth were seldom mentioned by elderly respondents. Perhaps the major indicator of the resilience of the elderly cancer patient is the equanimity with which they pursue their lives in the face of cancer. The distinctive nature of coping during the survivorship phase allowed for creating a typology of strategies.

The most commonly mentioned coping strategy included eliciting social support, such as turning to others for emotional or instrumental support (40 responses). We call this group “The Networkers”. Response examples in this category include: “My wife was supportive and I am still trying to get help from my doctors”. While being a networker implies proactive efforts at social integration there was a subgroup of more dependent help seekers whom we also include in this category. This orientation is reflected in the response “I moved in with my daughter, she knows what to do”.

We label the next most common coping strategy as the “Take Charge Patients” (25 responses). These respondents revealed instrumental coping strategies whereby they took proactive steps to manage their illness. Examples of respondents’ sentiments include: “I chose self-reliance, if you have a problem, take care of it” and “I consistently kept doctor appointments, I tried an organic diet, read pertinent articles and shared my experiences with others.”

Another coping strategy that emerged from the patient responses was religious coping (24 responses). These “Pious Copers” turned to praying or spirituality during the survivorship phase. Examples of responses of these copers included: “I used my spiritual self to cope” and “I had a belief God was looking after me and he did. Religion and spirituality, prayer has been a big help.”

Some respondents reported intra-psychic coping strategies that included a reappraisal of the situation and making positive comparisons. We call these respondents the “Stay Positive Patients” (23 responses). Examples of these responses include: “Acceptance helped me by seeking the inspiration and consolation. I kept a positive attitude” and “Forgetting about it and live your life every day and just be positive.”

We refer to the next category of coping as the “Passers By” (23 responses). These respondents felt that there was no need to enact coping strategies as the treatment they received cured them of their cancer. Examples include: “he cut it out and that was it” and “the cancer was eliminated and I led my life as it should.” This group also included patients who went on with their lives and chose not to dwell on their cancer experience. They realized that they were living with cancer, but made a conscious decision not to focus on their illness. Examples include: “There was no coping, I did what had to be done and that’s it” and “I have inner strength, I am a fatalist, what will be”.

The “Busy Bees” enacted coping strategies that reflected “keeping busy” and keeping one’s mind off of the challenges of the cancer experience (14 responses). Examples include: “Keeping up routines as much as possible and continuing to focus on other areas of my life as well” and “It wasn’t a challenge for me I didn’t have time… I had teenagers at home.”

A much smaller group of respondents reported that they engaged in emotional coping with a negative valence. We coded this category as the “Emotionally Afflicted” (3 responses). They became very sad in response to their cancer. One respondent noted, “I was angry in a way,” while another said “I cried a lot.”

Our final small group reported coping strategies of post-traumatic growth or transformation (3 responses). We referred to this category as the “Benefit Finders.” Examples include, “Some priorities have changed —you know what is important.” This group is particularly noteworthy due to the prominence of this mode of coping in the literature coupled with their relative absence in spontaneous responses in our sample.

## Discussion

The purpose of this study was to explore the meaning of living with cancer and older adults’ orientations to coping with stressors encountered during their cancer journey as reflected in narratives of elderly cancer survivors. Our results support prior findings regarding the potential protective influence of old age in responding to a diagnosis of life threatening illness [22]. The “on time” interpretation by some of the older adults of having a cancer diagnosis in later life may diminish the stressfulness of the diagnosis and may enhance the patients’ ability to proactively cope with the reality of their illness. Older adults may not have to grapple with disruption of role obligations that pose critical challenges during younger years [32]. Thus, nearing the end of a natural lifespan may facilitate acceptance of a cancer diagnosis with greater equanimity. Our findings also underscore older adults’ future orientation and “will to live” reflected in the proactive determination to fight their cancer [33]. As older patients come to inhabit the “lived world of knowing” their cancer diagnosis, most strive to normalize their lives [34] by rejection of a “spoiled identity” [35] and through proactive coping strategies that are the hallmark of successful aging [36].

Our previously developed a conceptual framework of targeted cancer adaptations reflects the recognition that unique coping responses must be tailored for the patient who receives a cancer diagnosis to meet adaptive tasks in multiple domains: 1) cognitive regulation; 2) emotion regulation; 3) social support regulation; 4) proactive illness management and 5) social role management [15]. According to our model, the older person diagnosed with cancer is called upon to ensure access to medical care, to gather health information, and to take initiative in communicating with the healthcare providers. Additionally, the patient diagnosed with cancer must seek state-of-the-art treatment and adhere to medical regimens involved in the treatment. Furthermore, patients also benefit from engaging in health promoting behaviors and form a comfortable healthcare environment. It can be readily seen that these targeted and proactive adaptations are far more specific than the traditionally broad coping categories of active and passive coping or of instrumental, intra-psychic and avoidant coping strategies discussed in the psychological literature [4].

Our data reveals noteworthy similarities as well as some differences among coping strategies articulated by elders regarding different phases of their cancer experience. Accordingly, respondents portrayed themes of competent coping and an ability to put their illness in perspective throughout their cancer journey. Differences portrayed in coping strategies relative to different phases of illness are appropriate to the adaptive tasks relevant during that phase of the cancer journey [37]. During the diagnosis phase there is a focus on fighting spirit and resolve to beat this threatening illness, whereas during the treatment phase, elderly patients tend to put their trust in their physicians and elicit social support from significant family and friends. Responses suggest that patients may be more passive as they focus on care getting during this acute illness phase [15]. Given the stressful nature of cancer treatments ranging from surgery to chemotherapy or radiation this propensity to adopt the sick role, seek competent help and relinquish other responsibilities is well-suited to this situation [33].

During the longer term survivorship phase older adults can look back at the adaptations they deem most useful. The majority of respondents reported that they value active coping styles. These include proactive care getting, or seeking social support as well as instrumental or active orientations to dealing with the illness experience. It is notable that the third most common coping strategy refers to religious or spiritual approaches that have been found to facilitate healing and finding meaning in prior research [24]. It may be argued that older adults employ successful behavioral, social and psychological maneuvers to maintain the integrity of the self during their cancer journey.

The types of adaptations described by our respondents as most helpful in managing their cancer journey in some respects map well with traditional conceptualizations of coping. The eight categories that emerged from our data include instrumental, intra-psychic, and escape oriented coping orientations. We also found evidence of focus by elderly cancer patients on hope-fostering coping strategies throughout their cancer journey [38]. This orientation is also consistent with prior work documenting the value of religion and spirituality in coping with cancer [39]. In spite of methodological limitations of prior studies focused on religious and spiritual coping such strategies can yield benefits based on increased self- esteem, a sense of meaning, and through offering emotional comfort [40].

Beyond confirming the salience of traditional definitions of coping, our qualitative approaches afford the opportunity to paint a more textured portrait that reveals the landscape of older adults’ encounter with cancer. It appears that the resilience acquired during a long life of facing and mastering stressful situations inoculates the elderly so that they can cope competently and retain a strong sense of self in spite of the threats posed by cancer. Themes shared by respondents reflect mastery throughout the cancer experience rather than posttraumatic growth as a function of cancer related trauma.

As we consider clinical implications of our findings we recommend that in treating older the patients diagnosed with cancer primary care physicians and oncologists should not only pay attention to vulnerabilities of aging, but also build on the special strengths that are evident at this late stage of the life course that enables elders to normalize their lives in spite of challenges of the cancer diagnosis.
